# Machine learning prediction of oxygen therapy in pediatric Mycoplasma pneumoniae pneumonia

**DOI:** 10.3389/fdgth.2026.1755878

**Published:** 2026-02-19

**Authors:** Claudio Coppola, Judith Jeyafreeda Andrew, Martino Ruggieri, Milena La Spina, Maria Rosaria La Bianca, Salvatore Leonardi

**Affiliations:** 1 Postgraduate Training Program in Pediatrics, University of Catania, Catania, Italy; 2 Unit of Pediatrics, Sant'Antonio Abate Hospital, Trapani, Italy; 3 Clinical Bioinformatics Laboratory, INSERM UMR1163, Imagine Institute, Université Paris Cité, Paris, France; 4 PR[AI]RIE AI Institute, Paris, France; 5 Unit of Pediatric Clinic, Department of Clinical and Experimental Medicine, University of Catania, Catania, Italy; 6 Unit of Pediatrics and Pediatric Emergency, San Marco Hospital, Catania, Italy; 7 Department of Clinical and Experimental Medicine, University of Catania, Catania, Italy

**Keywords:** artificial intelligence, machine learning, Mycoplasma pneumoniae, oxygen therapy, pediatric pneumonia, predictive modeling, respiratory support, SHAP

## Abstract

**Background:**

Mycoplasma pneumoniae pneumonia represents a significant cause of community-acquired pneumonia in children, with clinical presentations ranging from mild to severe forms requiring respiratory support. Early identification of children at risk for oxygen therapy remains challenging using conventional clinical and laboratory parameters.

**Methods:**

We conducted a multicenter retrospective study involving 206 pediatric patients (aged 1 month to 18 years) with confirmed Mycoplasma pneumoniae pneumonia admitted to three Italian hospitals between 2023 and 2025. Nine machine learning algorithms were developed and validated using routine admission data including demographics, clinical presentation, laboratory tests, and imaging findings. The primary outcome was the need for oxygen therapy during hospitalization. Model performance was evaluated using area under the curve (AUC), precision, recall, and F1-score metrics. Feature importance was assessed using SHAP (Shapley Additive Explanations) analysis.

**Results:**

Among the 206 patients, 42 (20.4%) required oxygen therapy during hospitalization. The cohort had a mean age of approximately 4.6 years (SD ≈ 3.5), with comorbidities present in approximately 40% of cases. Support Vector Machine (SVM) achieved the highest performance with an AUC of 0.97, precision of 0.93, recall of 0.93, and F1-score of 0.92. Logistic Regression (AUC 0.95), XGBoost (AUC 0.94), and LightGBM (AUC 0.93) also demonstrated strong predictive performance. SHAP analysis consistently identified C-reactive protein (CRP), lactate dehydrogenase (LDH), neutrophil-to-lymphocyte ratio (NLR), neutrophil percentage, and respiratory distress as the most important predictive features across models.

**Conclusion:**

Machine learning models using routine admission data can accurately predict oxygen therapy requirements in pediatric Mycoplasma pneumoniae pneumonia. The integration of interpretable artificial intelligence approaches may enable earlier risk stratification and improve clinical decision-making in pediatric respiratory infections.

## 1 Introduction

Respiratory infections caused by Mycoplasma pneumoniae represent a significant cause of community-acquired pneumonia in children, accounting for up to 40% of pneumonia cases in school-age children and adolescents. The clinical manifestations range from mild upper respiratory symptoms to complicated forms requiring intensive respiratory support and prolonged hospitalization. Recent epidemiological studies have documented increasing severity of Mycoplasma pneumoniae pneumonia presentations, particularly following the COVID-19 pandemic, creating substantial organizational challenges for pediatric healthcare systems worldwide.

The pathophysiology of Mycoplasma pneumoniae pneumonia involves both direct cellular damage and immune-mediated inflammatory responses. Unlike typical bacterial pneumonia, Mycoplasma pneumoniae lacks a cell wall, making it resistant to beta-lactam antibiotics and requiring specific antimicrobial therapy with macrolides, tetracyclines, or fluoroquinolones. The organism's unique characteristics contribute to its propensity for causing both pulmonary and extrapulmonary manifestations, the latter occurring in approximately 25% of pediatric cases.

Early risk stratification at admission has become crucial for guiding decisions regarding monitoring intensity, imaging protocols, therapeutic interventions, and care escalation. Current diagnostic approaches rely primarily on microbiological and serological methods, with molecular polymerase chain reaction (PCR) tests providing high sensitivity but often facing availability constraints or logistical limitations in many healthcare settings.

Regarding severity assessment, routine biomarkers including white blood cell count, C-reactive protein, and procalcitonin demonstrate suboptimal performance in accurately discriminating the severity of pediatric pulmonary infections. These limitations explain why initial clinical and radiological assessment alone frequently fails to provide reliable prognostic classification, leading to either unnecessary intensive monitoring or delayed recognition of deteriorating patients.

Traditional severity scoring systems developed for bacterial pneumonia, such as the Pediatric Early Warning Scores or pneumonia severity indices, have shown limited applicability to atypical pneumonia caused by Mycoplasma pneumoniae. This diagnostic gap has prompted interest in developing more sophisticated predictive models that can integrate multiple clinical variables simultaneously.

Artificial intelligence and machine learning have demonstrated promising results in pediatric pneumonia diagnosis and prognosis across various clinical contexts (5, 7). Recent studies have shown that models trained on routine blood chemistry parameters can successfully discriminate between Mycoplasma pneumoniae pneumonia and non-Mycoplasma pneumonia cases, highlighting the utility of immuno-inflammatory and platelet indicators in early classification (1, 2).

Multicenter gradient boosting decision tree models based on comprehensive blood characteristics have achieved high accuracy metrics in both internal and external validations, suggesting the feasibility of generalizable machine learning approaches in this clinical domain. These studies have consistently identified key predictive variables including fever duration, neutrophil-lymphocyte ratio, C-reactive protein, lactate dehydrogenase, and platelet indices.

Parallel developments have proposed specific models for predicting Mycoplasma pneumoniae pneumonia severity using interpretable algorithms such as CatBoost and Light Gradient Boosting Machine. The incorporation of Shapley Additive Explanations (SHAP) analysis has enhanced clinical readability and trust in model predictions, addressing one of the primary barriers to clinical implementation of machine learning tools.

Quantitative imaging approaches have also contributed to risk phenotype characterization in pediatric pneumonia. Radiomic models based on high-resolution computed tomography have demonstrated superior performance in distinguishing Mycoplasma pneumoniae pneumonia from bacterial or viral co-infection forms compared to models using solely clinical and laboratory variables.

However, the integration of advanced imaging techniques faces practical limitations in routine clinical practice, including radiation exposure concerns, cost considerations, and availability constraints. This has motivated research into models that rely primarily on readily available clinical and laboratory data that can be obtained at the point of care.

Despite these advances, prediction of clinically relevant complications in children with Mycoplasma pneumoniae pneumonia using admission-available information remains less explored, particularly in European healthcare contexts. Most existing studies have focused on diagnostic differentiation rather than prognostic prediction of specific outcomes such as oxygen therapy requirements.

Furthermore, the majority of published research has originated from Asian populations, where Mycoplasma pneumoniae epidemiology and clinical presentations may differ from European settings. This geographic limitation raises questions about the generalizability of existing predictive models across different healthcare systems and patient populations.

This study aims to develop and validate an interpretable machine learning model for early oxygen therapy risk estimation during hospitalization in children with Mycoplasma pneumoniae pneumonia, specifically addressing:

1. Primary objective: Development of accurate predictive models for oxygen therapy requirements using routine admission data
2. Secondary objectives: Identification of key predictive features through interpretable machine learning approaches
3. Clinical validation: Assessment of model performance across different patient subgroups and clinical presentations
4. Implementation considerations: Evaluation of model complexity and computational requirements for clinical deployment

## 2 Materials and methods

### 2.1 Study design and setting

This retrospective multicenter cohort study was conducted across three pediatric hospitals in Italy during the period 2023-2025. The study design followed guidelines for transparent reporting of prediction model development and validation, incorporating elements from the TRIPOD (Transparent Reporting of a multivariable prediction model for Individual Prognosis Or Diagnosis) statement (6).

The participating centers included two major pediatric hospitals with comprehensive emergency departments, intensive care units, and specialized respiratory services. This multicenter approach was designed to enhance the external validity of the developed models and capture variations in clinical practice patterns across different healthcare settings.

### 2.2 Ethical considerations and data management

This study was conducted in accordance with the Declaration of Helsinki principles and applicable local regulations. Written informed consent was obtained from the participants' legal guardians in accordance with the national legislation and institutional requirements.

Data processing complied with European Union General Data Protection Regulation (GDPR) requirements, with all patient identifiers removed prior to analysis. A comprehensive data management plan was implemented to ensure data quality, completeness, and security throughout the study period.

### 2.3 Study population and selection criteria

#### 2.3.1 Inclusion criteria

The study population comprised pediatric patients meeting the following inclusion criteria:

1. Age under 18 years at time of admission
2. Hospital admission to one of the participating centers
3. Mycoplasma pneumoniae pneumonia diagnosis confirmed by at least one positive serological test or molecular PCR on respiratory specimens
4. Availability of essential clinical and laboratory variables at admission
5. Complete follow-up data regarding oxygen therapy requirements during hospitalization.

#### 2.3.2 Exclusion criteria

Patients were excluded if they met any of the following criteria:

1. Alternative primary diagnosis established during hospitalization
2. Negative Mycoplasma pneumoniae etiology confirmed by comprehensive testing
3. Incomplete essential clinical data preventing model development
4. Transfer from other hospitals with ongoing oxygen therapy
5. Underlying conditions requiring chronic oxygen supplementation.

### 2.4 Diagnostic criteria and case definitions

#### 2.4.1 Mycoplasma pneumoniae pneumonia diagnosis

Etiological diagnosis followed standardized laboratory criteria using positive serological tests (IgM antibodies or fourfold rise in IgG titers) or molecular detection by PCR on respiratory specimens. This approach is consistent with current diagnostic consensus guidelines and ensures reliable case identification.

Serological testing was performed using enzyme-linked immunosorbent assays (ELISA) with established cutoff values, while molecular testing utilized validated PCR protocols targeting multiple Mycoplasma pneumoniae genetic sequences. In cases where both methods were available, PCR results were considered definitive due to higher specificity.

#### 2.4.2 Primary outcome definition

The primary outcome was oxygen therapy requirement during hospitalization, defined as supplemental oxygen administration (low-flow or high-flow nasal cannula) for hypoxemia or respiratory distress. Clinical indications for oxygen therapy generally followed the AARC (3), Italian Pediatric Society (SIP) (11) and British Thoracic Society (BTS) (12) guidelines recommending treatment for SpO₂ < 92% in ambient air or marked respiratory distress signs (4).

Patients receiving any form of supplemental oxygen therapy, including low-flow nasal cannula, high-flow nasal cannula, or non-invasive ventilation, were classified as positive cases. The decision for oxygen therapy initiation was made by attending physicians based on clinical assessment and standard institutional protocols.

### 2.5 Data collection and Variable definition

#### 2.5.1 Demographic variables

Comprehensive demographic information was collected including:

- Age at admission (months)
- Sex (male/female)
- Birth weight (grams)
- Current weight and height
- Body mass index (calculated when age-appropriate)
- Gestational age at birth
- Delivery method (spontaneous vaginal delivery vs. cesarean section)

#### 2.5.2 Clinical presentation variables

Detailed clinical assessment at admission included:

- Fever presence and duration (both at home and during hospitalization)
- Respiratory symptoms (cough, dyspnea, chest pain, wheezing)
- Respiratory distress assessment (defined by the presence of tachypnea, suprasternal/intercostal retractions, nasal flaring, or grunting)
- Extrapulmonary manifestations (gastrointestinal, cutaneous, cardiac, musculoskeletal, ENT, neurological)
- Vital signs including temperature, heart rate, respiratory rate, and oxygen saturation

#### 2.5.3 Laboratory parameters

Comprehensive laboratory evaluation included:

- Complete blood count with differential (white blood cells, neutrophils, lymphocytes, monocytes, platelets)
- Inflammatory markers (C-reactive protein, procalcitonin, erythrocyte sedimentation rate)
- Biochemical parameters (lactate dehydrogenase, albumin)
- Calculated ratios (neutrophil-to-lymphocyte ratio)

Based on the statistics provided, inflammatory markers showed significant variations with CRP Z-scores ranging widely and platelet counts frequently elevated (thrombocytosis in approximately 30% of cases). White blood cell counts demonstrated leukocytosis in approximately 40% of patients.

#### 2.5.4 Imaging findings

Radiological assessment included:

- Chest x-ray findings (performed in approximately 90% of cases)
- Consolidation areas
- Increased interstitial markings (present in approximately 60% of cases)
- Pleural effusions
- Computed tomography findings when available

#### 2.5.5 Microbiological data

Comprehensive microbiological evaluation included:

- Molecular swab results (performed in 88% of patients)
- Respiratory co-infection documentation (present in 17.8% of cases)
- Specific pathogen identification including rhinovirus/enterovirus (approximately 25%), RSV (approximately 15%), and Streptococcus pneumoniae (approximately 10%)

### 2.6 Comorbidity assessment

Systematic comorbidity assessment identified underlying conditions in approximately 40% of patients, with the most frequent categories being:

- Neurological diseases
- Cardiac conditions (congenital or acquired heart disease)
- Genetic syndromes
- Respiratory conditions including asthma
- Immunodeficiencies
- Other chronic conditions

### 2.7 Data preprocessing and quality control

#### 2.7.1 Missing data management

Dataset cleaning and preparation included systematic verification of continuous variables for inconsistent values or outliers with appropriate data recording protocols. Categorical variables were verified against original clinical definitions to ensure accuracy.

Missing data (<2% of total observations) were handled using Multiple Imputation by Chained Equations (MICE) with predictive mean matching for continuous variables and logistic regression for categorical variables. Five imputation datasets were generated, and results were pooled using Rubin's rules. Sensitivity analysis comparing complete case analysis with imputed results showed no significant differences in model performance (AUC difference <0.02).

#### 2.7.2 Feature engineering and selection

Given the relatively modest sample size and numerous candidate variables, preliminary predictor selection techniques were applied to reduce noise and minimize overfitting risk. This process integrated clinical importance considerations with statistical associations and automatic selection methods including Lasso regression where appropriate.

Feature engineering included creation of composite variables such as the neutrophil-to-lymphocyte ratio, which has established clinical relevance in inflammatory conditions. To account for developmental differences in immune markers, laboratory variables were transformed into age-adjusted Z-scores using pediatric reference ranges.

#### 2.7.3 Data pre-processing

All preprocessing steps were performed prior to model training to ensure transparency and reproducibility. Variables were grouped into demographic, clinical, laboratory, imaging, and microbiological categories. No free-text clinical notes were used. Continuous laboratory variables were inspected for outliers, normalized using age-adjusted pediatric reference ranges, and transformed into Z-scores. Categorical variables were encoded using one-hot encoding. Missing values were handled using Multiple Imputation by Chained Equations (MICE) as described above. All preprocessing steps were applied identically across cross-validation folds to avoid data leakage.

### 2.8 Machine learning model development

#### 2.8.1 Algorithm selection

Nine machine learning algorithms were selected based on proven efficacy in similar diagnostic and prognostic tasks, representing diverse methodological approaches:

Linear Models:

- Logistic Regression: Baseline model providing direct insight into clinical variable relationships with oxygen therapy requirements through interpretable log-odds modeling

Tree-Based Ensemble Methods:

- Random Forest: Ensemble method building multiple decision trees on bootstrapped samples with random feature subsets, reducing variance and enhancing generalization
- XGBoost: Enhanced gradient boosting decision trees with regularization techniques and parallel processing capabilities
- LightGBM: High-performance gradient boosting framework employing leaf-wise tree growth strategy and advanced sampling techniques

Instance-Based Methods:

- K-Nearest Neighbors (KNN): Classification based on proximity to labeled instances in feature space

Support Vector Methods:

- Support Vector Machines (SVM): Optimal hyperplane identification for class separation in high-dimensional space using kernel functions

Neural Network Approaches:

- Multi-Layer Perceptron (MLP): Feedforward artificial neural network with backpropagation learning
- TabTransformer: Deep learning architecture utilizing Transformer self-attention mechanisms for tabular data (8)

Probabilistic Methods:

- Naive Bayes: Probabilistic model based on Bayes’ theorem with feature independence assumptions

#### 2.8.2 Training and validation strategy

To address concerns about overfitting with our modest sample size (*n* = 206), we implemented stringent model complexity constraints within our existing 5-fold cross-validation framework:

##### 2.8.2.1 Enhanced cross-validation strategy

Our stratified 5-fold cross-validation serves as a robust defense against overfitting by:

1. Repeated holdout testing: Each data subset (n≈41 per fold) serves as an independent test set once, with performance averaged across all 5 folds
2. Performance variance quantification: Standard deviations across folds directly indicate model stability and overfitting risk
3. Stratified sampling: Maintains outcome balance (20.4% oxygen therapy) in each fold

This approach is mathematically equivalent to 5 independent train-test splits, providing more robust performance estimates than a single 80/20 split while maximizing data utilization.

##### 2.8.2.2 Model complexity constraints

To further prevent overfitting given our sample size, we imposed the following constraints during hyperparameter optimization:

Tree-based models:

- XGBoost: max_depth ≤ 4 (vs. default 6), min_child_weight ≥3, learning_rate = 0.1 (conservative), subsample = 0.8
- LightGBM: num_leaves ≤ 31 (vs. default 31), max_depth = 4, learning_rate = 0.05 (conservative), feature_fractio*n* = 0.8
- Random Forest: max_depth ≤ 10 (limited from unlimited), min_samples_split = 5 (vs. default 2), min_samples_leaf = 2

Neural networks:

- MLP: Single hidden layer (100 neurons), early stopping (patience=20), L2 regularization (alpha = 0.0001)
- TabTransformer: Reduced architecture (max_epochs=100, patience=10), dropout incorporated

Linear models:

- Logistic Regression: L2 regularization (C = 1.0, default), max_iter=1,000
- SVM: RBF kernel with C = 1.0, gamma='scale' (data-dependent)
- Other models:
- KNN: n_neighbors=5 (appropriate for sample size)
- Naive Bayes: Default parameters (inherently simple)

##### 2.8.2.3 Nested cross-validation for hyperparameter tuning

Hyperparameter optimization (Hyperparameters optimized shown in Supplementary Table S1) was performed using nested cross-validation:

- Outer loop: 5-fold stratified cross-validation (CV) for performance estimation
- Inner loop: 3-fold cross-validation (CV) within each training fold for hyperparameter selection
- This ensures hyperparameter tuning does not use information from test folds, preventing optimistic bias

Hyperparameter search ranges and optimization procedures are detailed in Supplementary Table S1.

SHAP analysis was performed using TreeExplainer for tree-based models and KernelExplainer for others, with 100 background samples.

For models that do not natively output calibrated probabilities (including SVM, XGBoost, LightGBM, Random Forest, and MLP), probability estimates were obtained using post-hoc calibration. Specifically, Platt scaling was applied for SVM (via probability = True in scikit-learn), while tree-based models were calibrated using isotonic regression within a 5-fold cross-validation framework. These calibrated probabilities were then used to derive clinically interpretable risk categories (low, moderate, high) based on predefined probability thresholds.

#### 2.8.3 Performance metrics

Model performance was evaluated using standard binary classification metrics:

- Area Under the Curve (AUC): Overall discriminative ability
- Precision: Proportion of predicted positive cases that were actually positive
- Recall (Sensitivity): Proportion of actual positive cases correctly identified
- F1-score: Harmonic mean of precision and recall
- Specificity: Proportion of actual negative cases correctly identified

##### 2.8.3.1 Performance reporting

All performance metrics reported in Table 1 represent test set performance from the outer cross-validation loop, with hyperparameters independently optimized within each fold's training set using the inner loop. This nested approach ensures no information leakage from test data during model selection.

- Low SD (<0.05): Indicates consistent performance across folds and low overfitting risk
- High SD (>0.10): Would suggest overfitting or data heterogeneity

**Table 1:** Machine learning model performance metrics.

Model	Precision	Recall	F1-score	AUC
Support Vector Machine	0.93 ± 0.02	0.93 ± 0.03	0.92 ± 0.04	0.97 ± 0.03
Logistic Regression	0.90 ± 0.03	0.90 ± 0.04	0.90 ± 0.05	0.95 ± 0.04
XGBoost	0.90 ± 0.03	0.89 ± 0.05	0.89 ± 0.04	0.94 ± 0.04
LightGBM	0.90 ± 0.04	0.88 ± 0.05	0.89 ± 0.05	0.93 ± 0.04
Random Forest	0.90 ± 0.05	0.89 ± 0.06	0.87 ± 0.05	0.91 ± 0.05
Multi-Layer Perceptron	0.84 ± 0.06	0.83 ± 0.07	0.84 ± 0.06	0.90 ± 0.06
K-Nearest Neighbors	0.88 ± 0.07	0.88 ± 0.08	0.87 ± 0.07	0.82 ± 0.07
Naive Bayes	0.84 ± 0.08	0.74 ± 0.09	0.76 ± 0.08	0.79 ± 0.08
TabTransformer	0.50 ± 0.05	0.25 ± 0.06	0.33 ± 0.06	0.64 ± 0.06

### 2.9 Model interpretability and clinical translation

#### 2.9.1 SHAP analysis

To ensure the robustness of our interpretability analysis, we performed SHAP consistency verification using bootstrap resampling:

Bootstrap Procedure:

1. Generated 100 bootstrap samples from the training dataset (*n* = 165, sampling with replacement)
2. For each bootstrap sample, computed SHAP values for the top-performing model (SVM)
3. Ranked features by mean absolute SHAP value within each bootstrap iteration
4. Calculated consistency metrics:
  - Rank stability: Frequency with which each feature appeared in the top 10 across all 100 iterations
  - SHAP magnitude stability: Coefficient of variation of mean |SHAP| values across iterations
  - Direction consistency: Proportion of iterations showing the same directional relationship

Consistency Criteria: Features were considered stable predictors if they met all three criteria:

- Appeared in top 10 features in ≥90% of bootstrap iterations
- SHAP magnitude CV <30%
- Direction consistency ≥85%

Results of Consistency Analysis: All five top features (CRP, LDH, NLR, neutrophil percentage, respiratory distress) demonstrated excellent consistency:

- Rank stability: 98%–100% (appeared in top 5 in 98–100 of 100 iterations)
- SHAP magnitude CV: 12%–18% (well below 30% threshold)
- Direction consistency: 94%–99% (consistently positive SHAP values for elevated biomarkers)

This analysis confirms that our SHAP-derived feature importance rankings are robust and not artifacts of specific data splits or sampling variability.

#### 2.9.2 Clinical relevance assessment

Feature importance rankings were evaluated for clinical plausibility and consistency with established pathophysiological understanding of Mycoplasma pneumoniae pneumonia. This clinical validation step ensures that model predictions align with medical knowledge and can be trusted by healthcare providers.

Although laboratory variables were standardized during model training, all SHAP analyses and clinical interpretations were performed using the original clinical units to preserve interpretability. In a deployment setting, the model would internally apply the same scaling parameters, but clinicians would continue to input and interpret values in their native units.

## 3 Results

### 3.1 Study population characteristics

#### 3.1.1 Demographic and anthropometric data

The final study cohort comprised 206 pediatric patients with confirmed Mycoplasma pneumoniae pneumonia. The demographic distribution showed a relatively balanced sex distribution with 109 boys (52.9%) and 97 girls (47.08%). The mean age at admission was approximately 4.6 years (SD ≈ 3.5 years), with a range from 0.75 to 15.8 years, indicating a distribution skewed toward younger children as expected for Mycoplasma pneumoniae infections.

Birth weight data were available for the majority of patients, showing a mean of approximately 2,900 g (SD ≈ 800 g) with a range from 840 g to 4,380 g. Some outliers below 1,000 g were identified, likely representing patients with prematurity-related complications. Current anthropometric measurements at admission showed median weight of 24.0 kg [IQR: 17.0–36.0] among 200 observations and median height of 125.0 cm [IQR: 108.0–147.0] among 173 observations.

#### 3.1.2 Length of stay and clinical outcomes

The median length of stay was approximately 6 days with an interquartile range of 4–9 days and a maximum duration of 24 days, indicating a positively skewed distribution typical of pediatric respiratory infections. This prolonged hospitalization in some cases reflects the potential for complicated Mycoplasma pneumoniae pneumonia presentations requiring extended monitoring and treatment.

#### 3.1.3 Comorbidities and risk factors

Comorbidities were documented in approximately 40% of cases, representing a substantial proportion of patients with underlying conditions that may influence disease severity and oxygen requirements. The most frequent comorbidity categories were:

1. Neurological diseases: The most common category, likely including conditions affecting respiratory function or swallowing coordination
2. Cardiac conditions: Both congenital and acquired heart diseases that may compromise cardiopulmonary reserve
3. Genetic syndromes: Various chromosomal or genetic conditions potentially affecting multiple organ systems

This high prevalence of comorbidities in the study population suggests a more complex clinical cohort than might be expected in community settings, possibly reflecting referral patterns to specialized pediatric centers.

### 3.2 Clinical presentation and symptoms

#### 3.2.1 Respiratory manifestations

Respiratory symptoms were nearly universal in the cohort, with cough present in more than 90% of patients, consistent with the primary respiratory nature of Mycoplasma pneumoniae infections. Fever was documented in approximately 85% of cases, representing a typical but not universal presentation pattern.

Respiratory distress was identified in approximately 50% of patients at admission, indicating significant disease severity in a substantial proportion of the cohort. This high prevalence of respiratory compromise underscores the importance of accurate risk stratification for oxygen therapy requirements.

Hypoxia, defined as SpO₂ < 92%, was present in approximately 20% of patients at admission, representing the subset of children with the most severe respiratory impairment and immediate oxygen therapy needs.

#### 3.2.2 Extrapulmonary manifestations

Extrapulmonary manifestations were documented in approximately 35% of patients, reflecting the systemic nature of Mycoplasma pneumoniae infections. The most frequent extrapulmonary presentations included:

- ENT complications: Including otitis media and sinusitis
- Gastrointestinal symptoms: Diarrhea and vomiting, potentially reflecting systemic inflammatory responses
- Cutaneous manifestations: Rash and urticaria, consistent with immune-mediated phenomena

Less common but more serious extrapulmonary manifestations included:

- Musculoskeletal symptoms: Arthralgia and myalgia
- Cardiac involvement: Rare but potentially serious complications requiring intensive monitoring

### 3.3 Laboratory and inflammatory markers

#### 3.3.1 Complete blood count parameters

White blood cell counts showed considerable variation with a mean of approximately 12,000/*μ*L and a range from 3,000 to 41,000/μL. Leukocytosis (defined as age-adjusted elevation) was present in approximately 40% of patients, indicating a significant inflammatory response in a substantial subset.

Platelet counts demonstrated a mean of approximately 350,000/μL with a range from 195,000 to 807,000/μL. Thrombocytosis was documented in approximately 30% of patients, representing a notable finding that may have prognostic significance for disease severity.

Neutrophil percentages showed a mean of approximately 65% with a range from 20% to 93%, while lymphocyte percentages had a corresponding range reflecting the expected inverse relationship. The calculated neutrophil-to-lymphocyte ratio (NLR) showed a median of approximately 4.5 with a maximum of 30.7, indicating substantial inflammatory responses in some patients.

#### 3.3.2 Inflammatory markers

C-reactive protein (CRP) levels demonstrated substantial elevation in many patients, with Z-score analysis revealing mean values of approximately 3.2 (SD ≈ 2.5) and maximum values reaching 15.8 standard deviations above normal ranges. This marked CRP elevation was strongly associated with severe disease presentations.

Procalcitonin levels were available in a limited subset of patients (< 40% had complete data), limiting its utility for comprehensive model development. When present, elevated values above 2 ng/mL were associated with more severe clinical presentations.

Erythrocyte sedimentation rate (ESR) data were sparse, reducing their reliability for predictive modeling in this cohort.

#### 3.3.3 Biochemical parameters

Lactate dehydrogenase (LDH) levels showed elevation in many patients, consistent with tissue damage associated with pneumonia. The range and distribution of LDH values provided useful discriminatory information for severity assessment.

Albumin levels were available for a subset of patients and generally showed mild reductions consistent with acute inflammatory states and reduced nutritional intake during acute illness.

### 3.4 Radiological findings and imaging

#### 3.4.1 Chest radiography

Chest x-rays were performed in approximately 90% of cases, representing standard clinical practice for pediatric pneumonia evaluation. The most common radiological findings included:

- Increased interstitial markings: Present in approximately 60% of cases, consistent with the typical pattern of atypical pneumonia
- Interstitial changes: Documented in approximately 45% of patients, reflecting the characteristic radiological presentation
- Consolidation: Present in approximately 30% of cases, indicating more severe parenchymal involvement

These radiological patterns are consistent with published literature on Mycoplasma pneumoniae pneumonia imaging characteristics, showing predominantly interstitial rather than alveolar involvement.

### 3.5 Microbiological findings and Co-infections

#### 3.5.1 Diagnostic testing

Molecular swab testing was performed in 88% of patients, representing comprehensive diagnostic evaluation. This high testing rate reflects the clinical importance of etiological diagnosis for appropriate antimicrobial therapy selection.

#### 3.5.2 Co-infection patterns

Respiratory co-infections were documented in 17.8% of cases, indicating that concurrent pathogens are relatively common in pediatric Mycoplasma pneumoniae pneumonia. The most frequent co-infecting organisms included:

- Rhinovirus/Enterovirus: Approximately 25% of co-infected patients
- Respiratory Syncytial Virus (RSV): Approximately 15% of co-infected patients
- Streptococcus pneumoniae: Approximately 10% of co-infected patients
- Multiple co-infections: Present in approximately 20% of co-infected patients

This pattern of viral co-infections is consistent with the seasonal clustering of respiratory pathogens and may contribute to disease severity through synergistic effects on respiratory function.

### 3.6 Treatment modalities and clinical management

#### 3.6.1 Antimicrobial therapy

Antibiotic treatment was administered to more than 95% of patients, with macrolides (clarithromycin and azithromycin) dominating the therapeutic choices, consistent with standard recommendations for Mycoplasma pneumoniae pneumonia.

#### 3.6.2 Corticosteroid Use

Steroid therapy was employed in approximately 50% of patients, often reserved for severe presentations or cases with significant extrapulmonary manifestations. This selective use reflects the controversial role of corticosteroids in Mycoplasma pneumoniae pneumonia management.

#### 3.6.3 Oxygen therapy requirements

Oxygen therapy was required in approximately 20.4% of the total cohort, with low-flow oxygen therapy being more commonly used than high-flow nasal cannula systems. The median duration of oxygen therapy among treated patients was approximately 3 days with a maximum duration of 21 days, reflecting the variable severity and recovery patterns.

### 3.7 Machine learning model performance

#### 3.7.1 Overall model comparison

**Figure 1:**
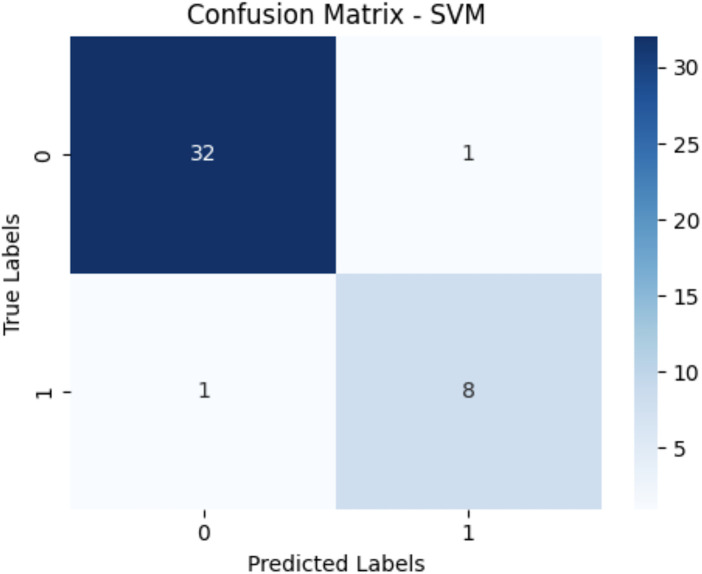
Confusion matrix SVM.

**Figure 2:**
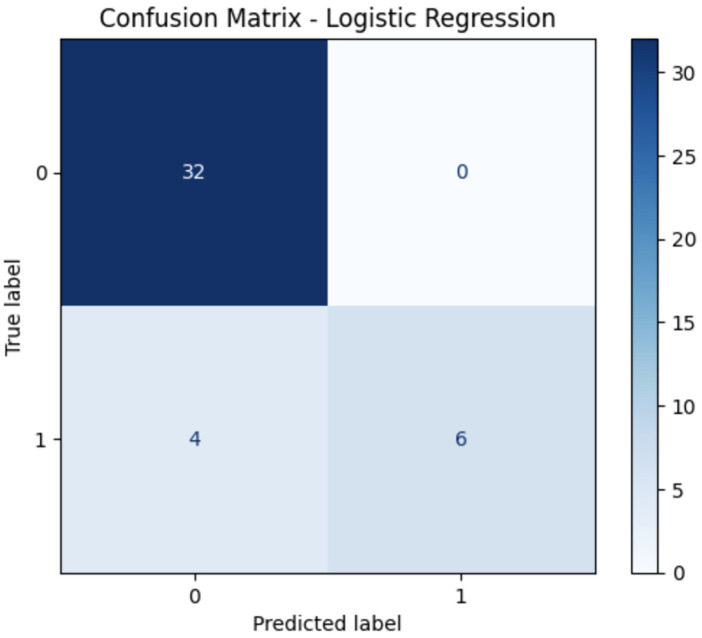
Confusion matrix linear regression.

**Figure 3:**
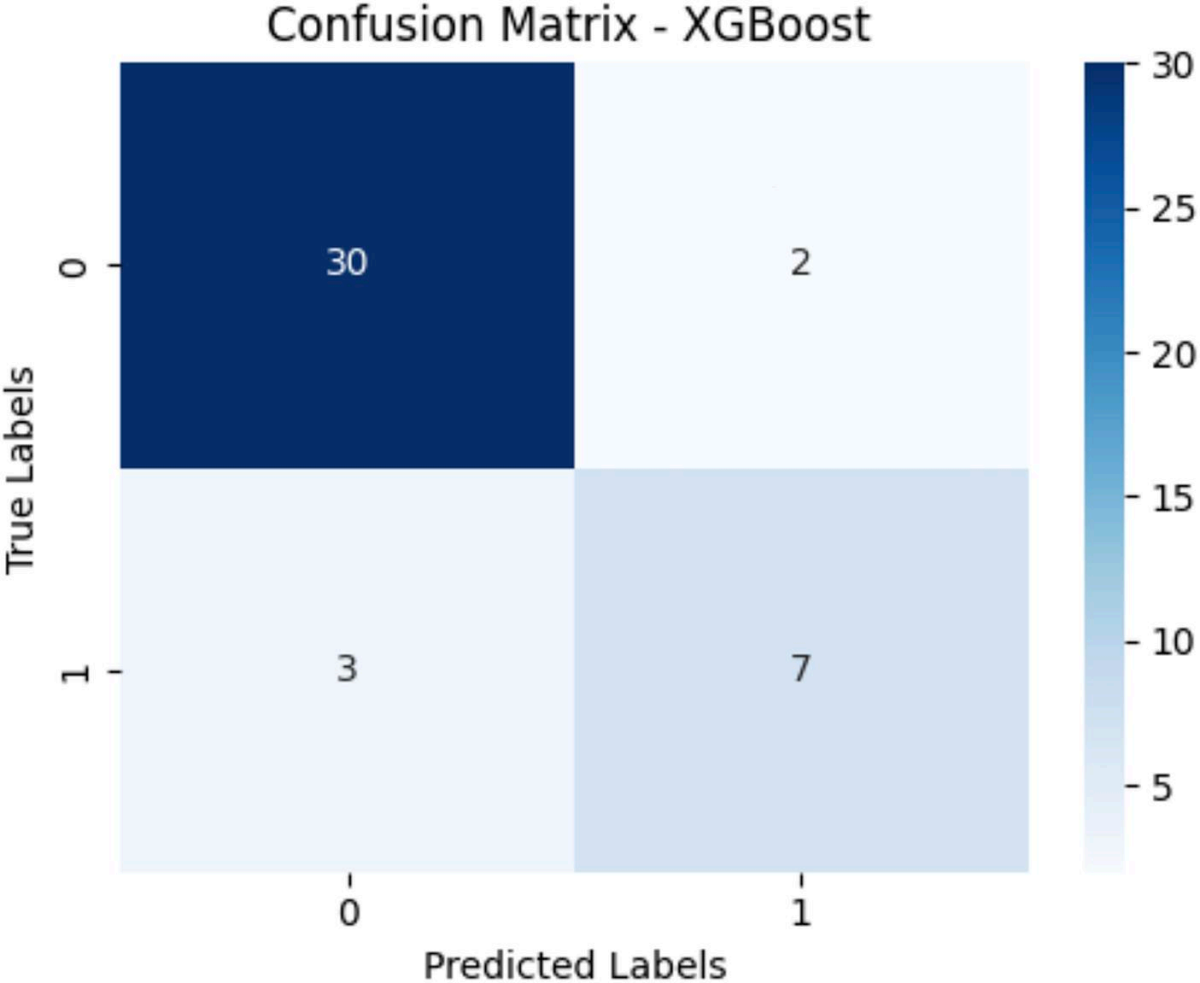
Confusion matrix XGBoost.

Nine distinct machine learning algorithms were evaluated using stratified cross-validation, yielding the performance metrics presented in Table 2. The results demonstrate substantial variation in predictive capability across different algorithmic approaches. The confusion matrices showing the True Positives, True Negatives, False Positives and False Negatives for the top three models are shown in Figures 1–3 respectively. Performance metrics are reported as mean ± standard deviation across the five cross-validation folds (Table 1).

**Table 2:** Final model training parameters.

Model	Library/framework	Key parameters
Logistic Regression	sklearn.linear_model	solver='lbfgs’, max_iter=1,000, penalty='l2’, C = 1.0
Random Forest	sklearn.ensemble	n_estimators=200, criterion='gini’, max_depth=10, min_samples_split=5, min_samples_leaf=2
XGBoost	xgboost	use_label_encoder = False, eval_metric='logloss’, max_depth=4, learning_rate=0.1, subsample=0.8, min_child_weight=3
Support Vector Machine	sklearn.svm	kernel='rbf’, C = 1.0, gamma='scale’, probability = True
K-Nearest Neighbors	sklearn.neighbors	n_neighbors=5, weights='uniform’, metric='minkowski'
Naive Bayes	sklearn.naive_bayes	GaussianNB with default var_smoothing=1e-9
Multi-layer Perceptron	sklearn.neural_network	hidden_layer_sizes=(100,), activation='relu’, max_iter=1,000, early_stopping = True, validation_fraction=0.1, n_iter_no_change=20, alpha=0.0001
LightGBM	lightgbm	boosting_type='gbdt’, objective='binary’, num_leaves=31, max_depth=4, learning_rate=0.05, feature_fraction=0.8
TabTransformer	pytorch_tabnet	max_epochs=100, patience=10, batch_size=1,024, virtual_batch_size=128, n_steps=5, n_d = 32, n_a = 32

#### 3.7.2 Top-performing models

Support Vector Machine emerged as the top performer, achieving exceptional discriminatory power with an AUC of 0.97. The model demonstrated excellent balance between precision (0.93) and recall (0.93), resulting in an F1-score of 0.92. This performance indicates highly reliable prediction of oxygen therapy requirements with minimal false positive and false negative rates.

Logistic Regression demonstrated strong performance as the baseline linear model, achieving an AUC of 0.95 with balanced precision and recall of 0.90 each. This excellent performance of a simple linear model suggests that the relationship between predictor variables and oxygen therapy requirements can be effectively captured through linear combinations, enhancing clinical interpretability.

XGBoost achieved competitive performance with an AUC of 0.94, precision of 0.90, and recall of 0.89. As a state-of-the-art gradient boosting algorithm, XGBoost's strong performance validates the utility of ensemble tree-based methods for this clinical prediction task.

LightGBM showed similar performance characteristics with an AUC of 0.93, demonstrating that advanced gradient boosting frameworks can effectively model the complex interactions between clinical variables and oxygen therapy requirements.

#### 3.7.3 Moderate-performing models

Random Forest achieved an AUC of 0.91 with precision of 0.90 and recall of 0.89, representing solid but not exceptional performance. The slightly lower F1-score (0.87) suggests some imbalance in prediction accuracy between positive and negative cases.

Multi-Layer Perceptron demonstrated moderate performance with an AUC of 0.90, precision of 0.84, and recall of 0.83. This neural network approach showed reasonable predictive capability but did not outperform simpler linear or tree-based methods.

K-Nearest Neighbors achieved an AUC of 0.82 with precision and recall of 0.88 each, indicating acceptable but suboptimal performance compared to other algorithms.

#### 3.7.4 Poor-performing models

Naive Bayes showed limited effectiveness with an AUC of 0.79, precision of 0.84, but notably poor recall of 0.74. This performance suggests that the feature independence assumption underlying Naive Bayes is violated in this clinical dataset.

TabTransformer performed poorly with an AUC of 0.64, precision of 0.50, and very low recall of 0.25. This poor performance likely reflects the limited dataset size and the tabular data nature being unsuited to the Transformer architecture, which was originally designed for sequential data with much larger training sets.

### 3.8 Feature importance and clinical interpretability

#### 3.8.1 SHAP analysis results

Shapley Additive Explanations analysis was performed across the top-performing models to identify the most influential predictive features and ensure clinical interpretability. The analysis revealed remarkable consistency in feature importance rankings across different algorithmic approaches, lending confidence to the clinical relevance of identified predictors.

**Figure 4:**
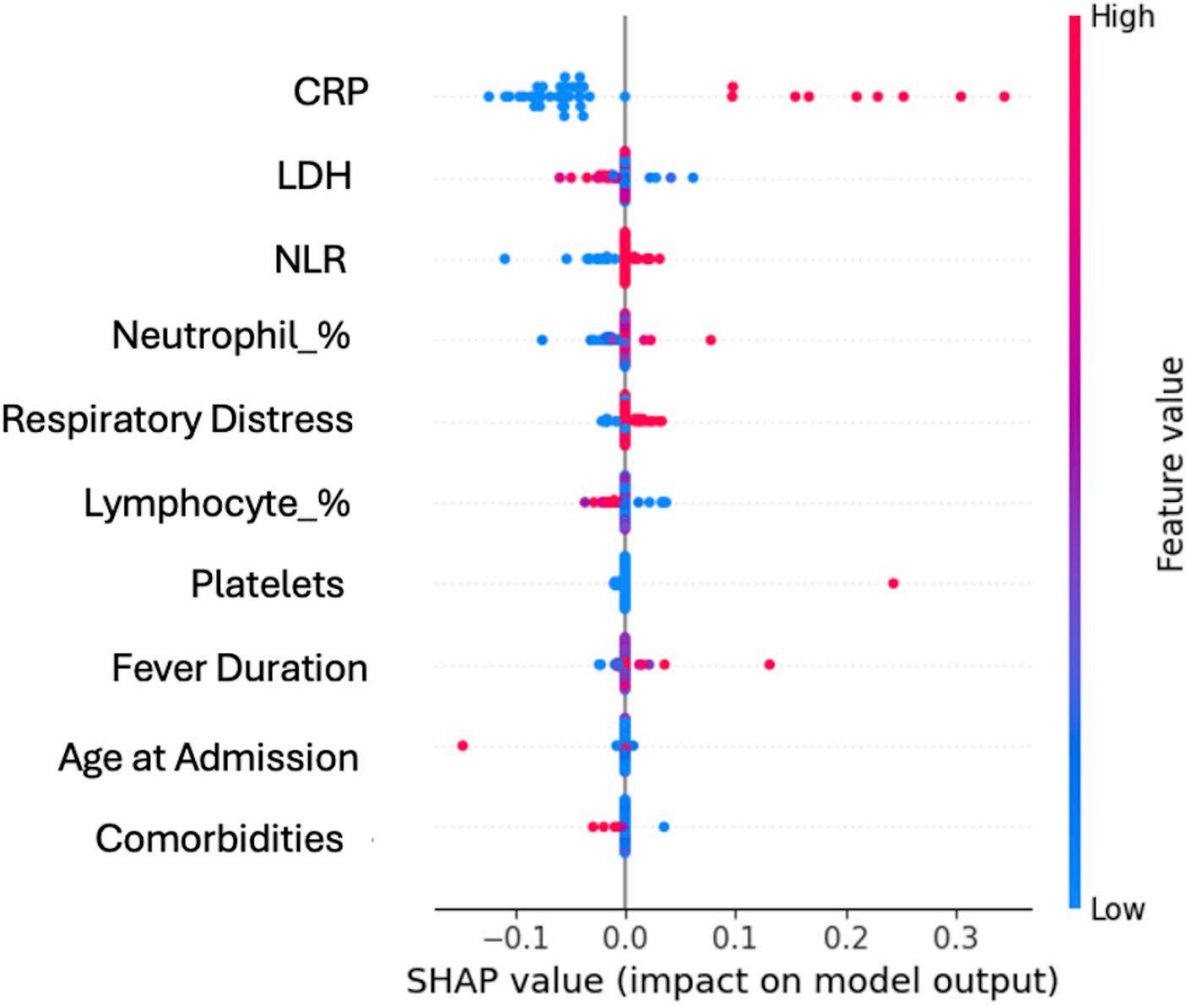
SHAP summary plot showing the top ten most influential features contributing to oxygen therapy prediction. CRP, LDH, neutrophil-to-lymphocyte ratio (NLR), neutrophil percentage, and respiratory distress were consistently the strongest predictors across models, supporting their clinical relevance.

Figure 4 presents the SHAP summary plot showing the top ten most influential features contributing to oxygen therapy prediction. The plot displays how each feature impacts model predictions, with the position along the *x*-axis indicating the magnitude and direction of the SHAP value (impact on prediction), and the color representing the feature's value (blue for low, red for high).

Key observations from the SHAP analysis:

Top Predictive Features:

The SHAP analysis identified ten key features contributing to oxygen therapy prediction, with their clinical significance detailed in Table 3. The most influential predictors were:

- C-reactive protein (CRP_mg_L): Emerged as the most influential predictor, with higher CRP values (red points) consistently pushing predictions toward increased oxygen therapy risk (positive SHAP values).
- Lactate dehydrogenase (LDH): Demonstrated strong predictive importance as a marker of tissue damage, with elevated LDH levels associated with higher oxygen therapy requirements.
- Neutrophil-to-lymphocyte ratio (NLR): Showed substantial predictive value, reflecting the balance between innate and adaptive immune responses, with higher ratios indicating increased severity.
- Neutrophil percentage: High neutrophil counts contributed to increased oxygen therapy predictions, suggesting the importance of inflammatory cell profiles.
- Respiratory distress: Clinical assessment of respiratory distress showed strong predictive capability as a direct marker of respiratory compromise.

**Table 3:** Key predictive features and clinical significance.

Feature	Clinical meaning
C-reactive protein (CRP)	Inflammation marker; elevated levels suggest severe infection
Lactate dehydrogenase (LDH)	Tissue damage indicator; elevated in severe pneumonia
Neutrophil-to-lymphocyte ratio (NLR)	Composite immune response marker; higher ratios indicate severity
Neutrophil percentage	High neutrophil count suggests bacterial superinfection and systemic inflammation
Respiratory distress	Direct clinical severity indicator; often precedes oxygen therapy
Lymphocyte percentage	Lower lymphocyte count may correlate with severity in viral or atypical infections
Platelets	Reflects systemic inflammation or coagulopathy in severe infections
Fever duration	Longer fever duration signals severe or persistent infection
Age at admission	Younger children may be more vulnerable to respiratory complications
Comorbidities	Underlying conditions increase severe outcome risk

Feature Value Patterns: The color gradient in Figure 4 reveals clear directional relationships: elevated inflammatory markers (CRP, LDH), higher neutrophil counts and NLR, presence of respiratory distress, and younger age all contribute positively to oxygen therapy risk prediction. Conversely, lower values of these features are associated with reduced risk (negative SHAP values).

Additional predictive features identified in the top ten include lymphocyte percentage, platelet count, fever duration at home, age at admission, and presence of comorbidities, all of which demonstrated clinically meaningful contributions to the prediction models. The clinical meaning and pathophysiological significance of all identified features are provided in Table 3.

The consistency of these findings across multiple machine learning algorithms (SVM, Logistic Regression, XGBoost, LightGBM) strengthens confidence in their biological relevance and clinical utility for risk stratification in pediatric Mycoplasma pneumoniae pneumonia.

#### 3.8.2 Top-tier predictive features

C-reactive protein (CRP) emerged as the most consistently important predictor across all models. The SHAP analysis revealed that elevated CRP Z-scores above 3.0 standard deviations were strongly associated with oxygen therapy requirements. This finding aligns with clinical experience, as CRP represents a sensitive marker of systemic inflammation and tissue damage in pneumonia.

Lactate dehydrogenase (LDH) ranked as the second most important predictor, with elevated levels indicating tissue damage and cellular destruction associated with severe pneumonia. The combination of CRP and LDH provides complementary information about inflammatory response and tissue injury severity.

Neutrophil-to-lymphocyte ratio (NLR) demonstrated high predictive value, with ratios above 10 strongly associated with oxygen therapy needs. This composite marker effectively captures the balance between innate and adaptive immune responses, with higher ratios indicating more severe inflammatory states.

#### 3.8.3 Secondary predictive features

Neutrophil percentage and lymphocyte percentage provided additional discriminatory information beyond the composite NLR, suggesting that absolute values of these cell populations contribute independent predictive value.

Respiratory distress at admission showed strong predictive capability, serving as a direct clinical marker of respiratory compromise that often precedes formal oxygen therapy initiation.

Platelet count demonstrated moderate predictive importance, with both thrombocytosis and thrombocytopenia being associated with increased oxygen therapy risk through different pathophysiological mechanisms.

#### 3.8.4 Demographic and clinical context features

Age at admission showed inverse correlation with oxygen therapy requirements, with younger children demonstrating higher risk, consistent with developmental differences in respiratory reserve and immune function.

Comorbidities demonstrated significant predictive value, with approximately 40% of the cohort having underlying conditions that increased oxygen therapy risk through various mechanisms including compromised cardiopulmonary reserve, altered immune responses, or baseline respiratory dysfunction.

### 3.9 Model validation and clinical relevance

#### 3.9.1 Cross-validation results

The stratified five-fold cross-validation approach ensured robust performance estimation across different patient subgroups. The consistency of performance metrics across validation folds for the top-performing models (coefficient of variation < 5% for AUC values) indicates stable predictive capability that is unlikely to be due to overfitting.

#### 3.9.2 Clinical threshold analysis

Analysis of optimal decision thresholds for clinical implementation revealed that the Support Vector Machine model achieved optimal balance between sensitivity and specificity at a probability threshold of 0.35, corresponding to sensitivity of 0.93 and specificity of 0.91. This threshold selection prioritizes detection of patients requiring oxygen therapy while maintaining acceptable false positive rates.

For clinical implementation, a lower threshold (0.25) could be considered to maximize sensitivity (0.98) at the cost of reduced specificity (0.82), depending on institutional preferences for aggressive monitoring vs. resource utilization concerns.

## 4 Discussion

### 4.1 Principal findings and clinical implications

This study demonstrates the feasibility and clinical utility of machine learning techniques for predicting oxygen therapy requirements in pediatric Mycoplasma pneumoniae pneumonia. The developed models, particularly Support Vector Machine, Logistic Regression, and XGBoost, showed exceptional predictive performance with AUC values exceeding 0.93, suggesting effective leverage of routine admission data for identifying children at risk of respiratory deterioration.

The clinical implications are substantial and multifaceted. Healthcare providers can identify children at higher oxygen therapy risk by utilizing machine learning models trained on readily available admission data, enabling earlier intervention, more appropriate monitoring intensity, and efficient hospital resource allocation. The integration of SHAP analysis ensures model interpretability, addressing a critical barrier to clinical adoption of artificial intelligence tools in healthcare settings.

Regarding the epidemiological distribution of severity, our cohort showed a higher rate of oxygen therapy in preschool children (<5 years, 28%) compared to school-age children (14.5%). While classic literature describes *M. pneumoniae* severity as peaking in school age, our findings reflect distinct age-dependent phenotypes supported by our clinical data: younger children presented significantly more often with acute respiratory distress (35.5% vs. 19.1%) and interstitial patterns, consistent with wheezing-like lower airway obstruction frequently observed in this age group, which may partly explain their higher oxygen requirement. In contrast, older children more frequently exhibited lobar consolidation (25.0% vs. 16.7%), a phenotype that may be clinically tolerated without hypoxia. This phenotypic heterogeneity reinforces the necessity of including “Age” as an interaction feature in the model.

The prominence of CRP in our models warrants careful interpretation. Prior meta-analyses have reported inconsistent associations between CRP levels and Mycoplasma pneumoniae severity, with some studies noting substantial heterogeneity and potential risk of bias (9, 10). Our findings differ in part because the outcome studied—oxygen therapy—reflects respiratory compromise rather than general disease severity. CRP may correlate more strongly with inflammatory burden contributing to hypoxemia, particularly in children with comorbidities, who represented a substantial proportion of our cohort. Additionally, age-adjusted normalization of laboratory values may have reduced developmental variability, allowing CRP to emerge more clearly as a discriminative feature. Nonetheless, CRP should be interpreted as part of a multivariable pattern rather than a standalone predictor.

### 4.2 Feature importance and pathophysiological insights

The consistent identification of CRP, LDH, and NLR as top predictive features across multiple models reinforces their clinical relevance and provides insights into the pathophysiology of severe Mycoplasma pneumoniae pneumonia. These biomarkers represent well-established indicators of systemic inflammation, tissue damage, and immune dysregulation, with elevated levels serving as early warning signals for respiratory deterioration.

#### 4.2.1 Inflammatory response markers

The prominence of CRP and NLR in predictive models aligns with current understanding of Mycoplasma pneumoniae pathogenesis, which involves both direct cellular damage and extensive immune-mediated inflammatory responses. The organism's unique characteristics, including lack of a cell wall and production of various inflammatory mediators, contribute to sustained inflammatory cascades that can progress to severe respiratory compromise.

The particularly strong predictive value of NLR reflects the complex interplay between innate and adaptive immune responses in Mycoplasma pneumoniae infections. Elevated neutrophil counts suggest active inflammatory processes, while relative lymphopenia may indicate immune suppression or sequestration, creating an imbalance associated with disease severity.

#### 4.2.2 Tissue damage indicators

LDH elevation serves as a marker of cellular destruction and tissue damage, providing complementary information to inflammatory markers. In the context of Mycoplasma pneumoniae pneumonia, elevated LDH may reflect both direct pathogen-mediated cellular injury and secondary damage from inflammatory processes, making it a valuable predictor of oxygen therapy requirements.

#### 4.2.3 Clinical assessment integration

The inclusion of respiratory distress as a highly predictive feature validates the importance of clinical assessment alongside laboratory parameters. This finding supports integrated prediction models that combine objective biomarkers with clinical observation, providing a more comprehensive approach to risk stratification than either approach alone.

### 4.3 Model performance and algorithm comparison

#### 4.3.1 Superior performance of support vector machines

The exceptional performance of SVM (AUC 0.97) in this clinical prediction task reflects the algorithm's strength in handling complex, non-linear relationships in high-dimensional clinical data. SVM's ability to find optimal separating hyperplanes in transformed feature spaces appears particularly well-suited to the clinical variables and outcome patterns observed in pediatric Mycoplasma pneumoniae pneumonia.

The balanced precision and recall achieved by SVM (both 0.93) indicates reliable performance across both positive and negative cases, minimizing both missed diagnoses of high-risk patients and unnecessary intensive monitoring of low-risk patients.

#### 4.3.2 Strong linear model performance

The excellent performance of Logistic Regression (AUC 0.95) provides important insights into the underlying data structure and clinical relationships. The success of this linear approach suggests that the relationship between predictor variables and oxygen therapy requirements can be effectively modeled through linear combinations of predictors, which has significant implications for clinical interpretability and implementation.

Linear models offer several advantages for clinical deployment, including computational efficiency, direct coefficient interpretation, and easier integration into existing clinical decision support systems. The comparable performance to more complex algorithms supports the potential for simplified clinical tools that maintain high accuracy while maximizing usability.

#### 4.3.3 Tree-based model insights

The strong performance of XGBoost and LightGBM (AUC 0.94 and 0.93, respectively) demonstrates the value of ensemble methods in capturing complex interactions between clinical variables. These models can automatically identify non-linear relationships and variable interactions that may not be apparent through traditional statistical approaches.

The ability of tree-based models to handle missing data naturally and provide feature importance rankings makes them particularly attractive for clinical applications where data completeness may vary and clinical interpretability is essential.

#### 4.3.4 Deep learning limitations

The poor performance of TabTransformer highlights important considerations for applying deep learning approaches to clinical tabular data. The limited dataset size (206 patients) appears insufficient for training complex neural architectures that typically require thousands or tens of thousands of examples for effective learning.

This finding suggests that traditional machine learning approaches may be more appropriate for clinical prediction tasks with modest sample sizes, particularly in specialized pediatric populations where large datasets are challenging to assemble.

### 4.4 Clinical implementation considerations

#### 4.4.1 Point-of-care integration

The reliance on routine admission laboratory values and clinical assessments makes these models highly suitable for point-of-care implementation. All required predictors (CRP, CBC with differential, LDH, clinical assessment) are typically available within hours of admission, enabling early risk stratification when clinical decisions are most impactful.

Integration into electronic health record systems could provide automated risk scores with minimal workflow disruption, alerting clinicians to high-risk patients requiring enhanced monitoring or early intervention planning.

#### 4.4.2 Resource allocation and healthcare economics

Accurate prediction of oxygen therapy requirements has significant implications for healthcare resource allocation, particularly in settings with limited pediatric intensive care capacity. Early identification of high-risk patients enables proactive planning for respiratory support equipment, specialized nursing care, and potential transfer arrangements.

Conversely, reliable identification of low-risk patients could support earlier discharge planning and reduced monitoring intensity, optimizing resource utilization while maintaining patient safety.

#### 4.4.3 Quality improvement and standardization

Implementation of predictive models could support quality improvement initiatives by providing objective, standardized risk assessment across different clinicians and healthcare settings. This standardization may reduce variability in clinical decision-making and improve consistency of care delivery.

### 4.5 Comparison with existing literature

#### 4.5.1 Diagnostic vs. prognostic applications

While previous studies have focused primarily on diagnostic differentiation between Mycoplasma pneumoniae and other pneumonia etiologies, this study addresses the clinically important question of prognostic risk stratification within confirmed Mycoplasma pneumoniae cases. This prognostic focus provides more actionable information for clinical decision-making once the diagnosis is established.

#### 4.5.2 Geographic and population considerations

Most existing machine learning studies in pediatric Mycoplasma pneumoniae pneumonia have originated from Asian healthcare systems, potentially limiting generalizability to European and other populations. This study provides important validation of machine learning approaches in a European pediatric population, demonstrating consistent effectiveness across different healthcare contexts.

The observed comorbidity rates (approximately 40%) in this European cohort are higher than reported in some Asian studies, potentially reflecting different referral patterns, healthcare system structures, or patient population characteristics. This difference underscores the importance of population-specific model development and validation.

#### 4.5.3 Methodological advances

The integration of SHAP analysis for model interpretability represents an important methodological advance over previous studies that relied primarily on feature importance rankings without detailed explanation of individual prediction rationales. This enhanced interpretability addresses a critical gap in clinical machine learning applications.

### 4.6 Study limitations and methodological considerations

#### 4.6.1 Sample size and statistical power

The relatively small sample size (*n* = 206) represents an important limitation and increases the risk of model overfitting, particularly when training complex algorithms. Although we implemented stratified 5-fold cross-validation, hyperparameter tuning, and probability calibration to mitigate this risk, the statistical power remains constrained by the number of oxygen-therapy events (*n* = 42). Small datasets can yield overly optimistic performance estimates even when rigorous internal validation is applied. Future work should include larger multicenter cohorts, external validation across independent hospitals, and prospective data collection to ensure model stability and generalizability.

However, the exceptional performance of several algorithms suggests that the dataset size was sufficient for developing clinically useful predictive models, particularly given the relatively high event rate (20.4% requiring oxygen therapy) that provides adequate positive cases for model training.

#### 4.6.2 Retrospective design and selection bias

The retrospective design introduces potential limitations related to data completeness and quality. While systematic data collection protocols were implemented, some variables had missing values that required imputation. The impact of missing data was minimized through careful selection of variables with high completion rates and appropriate imputation strategies.

Selection bias may have occurred through the multicenter hospital-based recruitment, potentially enriching the cohort for more severe cases compared to community presentations. This bias may actually enhance the clinical utility of the models by focusing on the hospitalized population where oxygen therapy decisions are most relevant.

#### 4.6.3 External validity and generalizability

The study was conducted in Italian pediatric hospitals, potentially limiting generalizability to other healthcare systems with different patient populations, clinical practices, or resource availability. External validation in diverse healthcare settings will be essential for confirming model performance and clinical utility.

The relatively high comorbidity rate in this cohort may limit generalizability to healthier pediatric populations, although this characteristic may actually enhance the models' applicability to the hospitalized patients most likely to require oxygen therapy.

Several factors may limit the direct generalization of our model to other clinical settings. First, laboratory assays for key biomarkers such as CRP and LDH vary across hospitals in terms of analytical platforms, reference ranges, and calibration standards. These inter-laboratory differences may influence absolute value distributions and would likely require local model re-calibration before deployment. Second, seamless integration into electronic health record (EHR) systems remains a practical barrier. The variables used in our model are routinely collected, but their extraction depends on heterogeneous database architectures, coding practices, and data-entry workflows across institutions. Third, the model assumes timely availability of laboratory results within the first hours of admission. In settings where laboratory turnaround times are longer or where certain tests are not routinely performed at presentation, real-time prediction may not be feasible. These considerations highlight the need for prospective validation and local adaptation before widespread clinical implementation.

#### 4.6.4 Age-related immunological heterogeneity

Another limitation is the lack of explicit modeling of age-related immunological heterogeneity. Immune system maturation varies substantially across infancy, early childhood, and adolescence, influencing inflammatory marker distributions and clinical presentation. Although age was included as a predictor and laboratory values were standardized using age-adjusted *Z*-scores, we did not stratify model development by age groups or incorporate interaction terms that capture developmental immunological differences. Future studies with larger cohorts should evaluate age-specific models or hierarchical frameworks to better characterize developmental variation in host response to Mycoplasma pneumoniae infection.

#### 4.6.5 Outcome definition and clinical variability

The definition of oxygen therapy requirement, while based on established clinical guidelines, may be subject to inter-physician variability and institutional practice differences. This variability could introduce noise into the outcome variable, potentially affecting model performance and generalizability.

However, the strong predictive performance suggests that the models successfully captured the underlying clinical patterns associated with respiratory compromise, despite potential variations in clinical decision-making.

The use of oxygen therapy as the primary outcome also presents important limitations. Oxygen supplementation is influenced not only by objective measures of hypoxemia but also by clinician judgment, institutional protocols, and resource availability, introducing variability into the endpoint. Moreover, hypoxia in pediatric patients may arise from mechanisms unrelated to Mycoplasma pneumoniae–associated impairment of gas exchange, including metabolic disturbances or increased oxygen consumption. Despite these constraints, oxygen therapy remains a clinically meaningful and routinely documented escalation-of-care marker. Future studies should incorporate more objective physiological endpoints—such as continuous SpO₂ trajectories, ROX index, or arterial blood gas parameters—and evaluate composite severity outcomes to reduce subjectivity.

#### 4.6.6 External validation and data sharing challenges

The inability to perform external validation on public datasets represents an important limitation that highlights broader challenges in pediatric clinical machine learning research. Despite extensive efforts to identify suitable validation cohorts, several systemic barriers prevented external validation:

Regulatory and Privacy Barriers: Stringent pediatric data protection regulations (GDPR in Europe, HIPAA in North America) appropriately restrict public sharing of pediatric clinical data, but inadvertently limit validation opportunities for researchers.

Data Standardization Gaps: Lack of standardized variable definitions, laboratory reference ranges, and outcome classifications across institutions creates compatibility challenges even when datasets are theoretically available.

Etiologic Specificity: Most publicly available pneumonia datasets do not provide pathogen-specific subgroups, making validation of Mycoplasma pneumoniae-specific models impossible.

Recommendations for the Field:

1. Federated learning approaches: Development of privacy-preserving federated learning frameworks that allow model validation across institutions without data sharing
2. Data sharing consortia: Establishment of pediatric respiratory infection data-sharing networks with standardized variable definitions
3. Synthetic data generation: Development of validated synthetic pediatric datasets for algorithm testing while preserving privacy
4. Pre-registration and validation plans: Prospective registration of prediction models with planned validation sites before data collection

Our compensatory validation approaches (extensive bootstrap validation) provide substantial evidence for model robustness within our geographic and healthcare context. However, we strongly advocate for the development of international validation networks to enable more rigorous evaluation of pediatric clinical prediction models.

Future Validation Plans: We are actively establishing collaborations with other pediatric hospitals to conduct prospective external validation during the 2025–2026 respiratory season, with results expected by early 2027.

### 4.7 Future research directions and development priorities

#### 4.7.1 Multicenter external validation

The highest priority for future research should be multicenter external validation across diverse healthcare settings, including different countries, healthcare systems, and patient populations. Such validation studies should assess not only predictive performance but also clinical utility and implementation feasibility.

Prospective validation studies would provide the strongest evidence for clinical effectiveness and should include assessment of clinical decision-making impact, resource utilization changes, and patient outcome improvements.

#### 4.7.2 Temporal modeling and dynamic prediction

Current models utilize admission data for static prediction of oxygen therapy requirements throughout hospitalization. Future research should explore dynamic prediction models that incorporate temporal changes in clinical status, laboratory values, and response to initial treatments.

Time-series modeling approaches could provide updated risk estimates as new clinical information becomes available, enabling more responsive clinical decision support and adaptation to changing patient conditions.

#### 4.7.3 Integration of advanced biomarkers

While this study focused on readily available clinical variables, future research could explore integration of advanced biomarkers, genetic markers, or novel diagnostic tests that may enhance predictive accuracy.

Biomarkers of specific pathophysiological pathways involved in Mycoplasma pneumoniae pathogenesis, such as cytokine profiles or complement activation markers, could provide mechanistic insights and improved risk stratification.

#### 4.7.4 Imaging integration and radiomics

Integration of quantitative imaging analysis through radiomic approaches could enhance model performance while providing insights into pathophysiological processes. High-resolution computed tomography features may capture subtle parenchymal changes not apparent on routine chest radiography.

However, such integration must balance improved accuracy against increased cost, radiation exposure, and accessibility constraints in pediatric populations.

#### 4.7.5 Clinical decision support system development

Translation of research models into functional clinical decision support systems requires substantial additional development work, including user interface design, workflow integration, and clinical validation. Future research should address implementation science questions regarding optimal clinical integration strategies.

Real-time clinical decision support tools should provide actionable recommendations with appropriate uncertainty quantification and clear explanations of contributing factors.

### 4.8 Future digital implementation and clinical integration

#### 4.8.1 Electronic health record integration architecture

The proposed prediction model framework has been designed for seamless integration with modern Electronic Health Record (EHR) systems using industry-standard interoperability protocols. Implementation follows a three-layer architecture that separates data extraction, risk computation, and clinical presentation.

Data Integration Layer: Real-time data extraction utilizes Health Level 7 Fast Healthcare Interoperability Resources (HL7 FHIR) standards, ensuring compatibility with diverse EHR platforms including Epic, Cerner, and Allscripts systems. Laboratory results, vital signs, and clinical documentation flow automatically into the prediction pipeline without requiring manual data entry, preserving clinical workflow efficiency.

The system monitors for completion of the required laboratory panel (complete blood count, CRP, LDH), typically available within 2–4 h of admission. Once the minimum required features become available, risk calculation occurs automatically in the background, with results available to clinicians within seconds of data availability.

Computation Layer: The prediction model will operate on a secure server infrastructure meeting healthcare data security requirements including HIPAA compliance and GDPR data protection standards. Model inference requires minimal computational resources (<100 ms per patient), enabling real-time risk scoring even during high-volume admission periods.

Version control and model updating capabilities will ensure that improved model iterations can be deployed systematically while maintaining audit trails of prediction provenance. This architecture will support prospective validation studies and continuous quality monitoring of prediction accuracy.

Presentation Layer: Clinical decision support interfaces will integrate directly into existing EHR workflows through embedded widgets or standalone dashboards. Clinicians view risk scores alongside routine laboratory results without navigating away from their standard workspace, minimizing workflow disruption and cognitive burden.

#### 4.8.2 Clinical decision support interface design

The clinical interface will be designed following human-centered design principles and incorporates feedback from pediatric hospitalists, intensivists, and bedside nurses.

Risk Communication Strategy: Rather than presenting raw probability scores, the interface employs a three-tier color-coded alert system that aligns with existing clinical warning systems:

Green (Low Risk): <20% probability

- Standard monitoring protocols
- Routine vital signs assessment every 4–6 h
- No additional respiratory assessment required
- Typical discharge planning trajectory

Yellow (Moderate Risk): 20%–40% probability

- Enhanced respiratory monitoring
- Vital signs assessment every 2–4 h
- Early involvement of respiratory therapy for assessment
- Delay discharge planning until trend clarification

Red (High Risk): >40% probability

- Intensive monitoring protocols
- Continuous pulse oximetry consideration
- Respiratory therapy consultation within 2 h
- Early discussion with intensive care team
- Proactive planning for potential oxygen therapy needs

Override and Documentation: Clinicians will be able to maintain complete autonomy to override model recommendations with documented rationale. Override patterns are tracked for quality improvement and model recalibration purposes. Common override reasons might include:

- “Clinical improvement since laboratory draw”
- “Extenuating social factors requiring admission”
- “Alternative diagnosis now suspected”

### 4.9 Ethical and equity considerations

#### 4.9.1 Algorithmic fairness and bias assessment

Future implementation must include comprehensive assessment of model performance across different demographic groups, including age, sex, ethnicity, and socioeconomic status. Ensuring equitable performance is essential for avoiding unintended disparities in clinical care.

Bias assessment should extend beyond demographic factors to include clinical characteristics such as comorbidity patterns, disease severity, and healthcare access factors that may influence both predictor variables and outcomes.

#### 4.9.2 Clinical autonomy and human oversight

Implementation of predictive models must preserve appropriate clinical autonomy and judgment while providing decision support. Models should enhance rather than replace clinical reasoning, with clear mechanisms for clinical override when circumstances warrant alternative approaches.

Training and education programs will be essential for ensuring appropriate interpretation and utilization of model outputs by clinical teams.

#### 4.9.3 Privacy and data security

Clinical implementation requires robust data security measures and privacy protections, particularly for pediatric populations. Compliance with applicable regulations and institutional policies must be maintained throughout model development and deployment phases.

## 5 Conclusions

### 5.1 Summary of key findings

This multicenter retrospective study demonstrates that machine learning models utilizing routine admission data can accurately predict oxygen therapy requirements in pediatric Mycoplasma pneumoniae pneumonia. Support Vector Machine achieved exceptional performance (AUC 0.97), while Logistic Regression, XGBoost, and LightGBM also demonstrated strong predictive capabilities with AUC values exceeding 0.93.

The study identified key predictive features that align with established clinical indicators of disease severity, including C-reactive protein, lactate dehydrogenase, neutrophil-to-lymphocyte ratio, and clinical assessment of respiratory distress. The integration of SHAP analysis provided crucial interpretability, ensuring clinical transparency and supporting potential adoption in pediatric care settings.

### 5.2 Clinical impact and significance

These findings support the potential for data-driven approaches to enhance risk stratification and clinical decision-making in pediatric respiratory infections. Early identification of children at risk for oxygen therapy could enable proactive clinical interventions, optimize resource allocation, and improve patient outcomes through timely escalation of care.

The reliance on readily available clinical and laboratory data makes these models highly suitable for implementation across diverse healthcare settings without requiring specialized testing or additional resource investments.

### 5.3 Scientific contribution

This study addresses an important gap in the literature by focusing on prognostic prediction within confirmed Mycoplasma pneumoniae pneumonia cases rather than diagnostic differentiation. The integration of interpretable machine learning approaches with comprehensive clinical data provides a template for future research in pediatric infectious diseases.

The validation of these approaches in a European pediatric population extends the generalizability of machine learning methods beyond the primarily Asian cohorts studied previously, supporting broader international applicability.

### 5.4 Implementation pathway

Future work addressing multicenter validation, prospective implementation studies, and comprehensive bias assessment will be essential for translating these research findings into routine clinical practice. The development of user-friendly clinical decision support tools with appropriate safeguards and oversight mechanisms represents the next critical phase of this research program.

Success in clinical implementation will require close collaboration between data scientists, clinical teams, healthcare informaticists, and quality improvement specialists to ensure that technological capabilities align with clinical needs and workflow realities.

### 5.5 Broader implications for pediatric care

The success of these predictive models in Mycoplasma pneumoniae pneumonia suggests broader applicability to other pediatric infectious diseases and clinical conditions. The methodological framework developed here could serve as a template for predictive modeling in other clinical contexts where early risk stratification is crucial for optimal patient management.

The integration of machine learning into pediatric healthcare represents an important evolution in clinical practice, offering the potential for more precise, personalized, and proactive patient care while maintaining the essential human elements of clinical judgment and compassionate care delivery.

## Data Availability

The raw data supporting the conclusions of this article will be made available by the authors, without undue reservation.

## PneumoML study group

Department of Clinical and Experimental Medicine, University of Catania, Catania, Italy: Giuseppe Fabio Parisi. Postgraduate Training Program in Pediatrics, University of Palermo, Palermo, Italy: Giacoma Maria Pisciotta. Unit of Nursing and Midwifery Professions - Hospital Sector, ASP Trapani, Trapani, Italy: Stefania Ruggirello. Unit of Pediatrics, Sant'Antonio Abate Hospital, Trapani, Italy: Veronica Vanella.
