# Supplementary material for: Machine learning prediction of oxygen therapy in pediatric Mycoplasma pneumoniae pneumonia
Source: Front Digit Health. 2026 Feb 19;8:1755878. doi: 10.3389/fdgth.2026.1755878 (PMC12960568; doi:10.3389/fdgth.2026.1755878)
Supplement: Supplementary file 1 [file Datasheet1.pdf]

TRIPOD Checklist: Prediction Model Development and Validation

Section	Item		Checklist description	Reported on Section/Paragraph
**Title and abstract**				
Title	1	D	Identify the study as developing and/or validating a multivariable prediction model, the target population, and the outcome to be predicted.	Title
Abstract	2	D	Provide a summary of objectives, study design, setting, participants, sample size, predictors, outcome, statistical analysis, results, and conclusions.	Abstract
**Introduction**				
Background and objectives	3a	D	Explain the medical context (including whether diagnostic or prognostic) and rationale for developing or validating the multivariable prediction model, including references to existing models.	Introduction, paragraph 1-3
	3b	D	Specify the objectives, including whether the study describes the development or validation of the model or both.	Introduction, last paragraph
**Methods**				
Source of data	4a	D	Describe the study design or source of data (e.g., randomized trial, cohort, or registry data), separately for the development and validation data sets, ifapplicable.	Section 2.1 "Study Design and Setting"
	4b	D	Specify the key study dates, including start of accrual; end of accrual; and, if applicable, end of follow-up.	Section 2.1 "Study Design and Setting"
Participants	5a	D	Specify key elements of the study setting (e.g., primary care, secondary care, general population) including number and location of centres.	Section 2.1 "Study Design and Setting"
	5b	D	Describe eligibility criteria for participants.	Section 2.3 "Inclusion Criteria" and "Exclusion Criteria"
	5c		Give details of treatments received, if relevant.	Section 3.6 "Treatment Modalities and Clinical Management"
Outcome	6a	D	Clearly define the outcome that is predicted by the prediction model, including how and when assessed.	Section 2.4.2 "Primary Outcome Definition"
	6b		Report any actions to blind assessment of the outcome to be predicted.	N/A - Retrospective study, outcome (oxygen therapy) was objective clinical intervention
Predictors	7a	D	Clearly define all predictors used in developing or validating the multivariable prediction model, including how and when they were measured.	Section 2.5 Data Collection and Variable Definition

	7b		Report any actions to blind assessment of predictors for the outcome and other predictors.	N/A - Retrospective study
Sample size	8	D	Explain how the study size was arrived at.	Section 3.7.4 Poor-Performing Models
Missing data	9	D	Describe how missing data were handled (e.g., complete-case analysis, single imputation, multiple imputation) with details of any imputation method.	Section 2.7.1 Missing Data Management
Statistical analysis methods	10a	D	Describe how predictors were handled in the analyses.	Section 2.7.2 Feature Engineering and Selection
	10b	D	Specify type of model, all model-building procedures (including any predictor selection), and method for internal validation.	Section 2.8 Machine Learning Model Development
	10c	V	For validation, describe how the predictions were calculated.	Section 2.8.2 Training and Validation Strategy
	10d	V	Specify all measures used to assess model performance and, if relevant, to compare multiple models.	Section 2.8.3 "Performance Metrics"
	10e		Describe any model updating (e.g., recalibration) arising from the validation, if done.	N/A - Development study only, no validation cohort requiring updating
Risk groups	11		Provide details on how risk groups were created, if done.	N/A - Study focused on binary prediction (oxygen therapy yes/no), not risk stratification into groups
Development vs. validation	12		For validation, identify any differences from the development data in setting, eligibility criteria, outcome, and predictors.	N/A - Single cohort study, no separate validation cohort
**Results**				
Participants	13a	D	Describe the flow of participants through the study, including the number of participants with and without the outcome and, if applicable, a summary of the follow-up time. A diagram may be helpful.	Section 3.1.1
	13b	D	Describe the characteristics of the participants (basic demographics, clinical features, available predictors), including the number of participants with missing data for predictors and outcome.	Sections 3.1-3.5
	13c		For validation, show a comparison with the development data of the distribution of important variables (demographics, predictors and outcome).	N/A - No separate validation cohort
Model development	14a	D	Specify the number of participants and outcome events in each analysis.	Section3.1.1

	14b		If done, report the unadjusted association between each candidate predictor and outcome.	N/A - Machine learning approach, not traditional regression
Model specification	15a	D	Present the full prediction model to allow predictions for individuals (i.e., all regression coefficients, and model intercept or baseline survival at a given time point).	Table 1
	15b	D;V	Explain how to the use the prediction model.	Section 4.4.1 "Point-of-Care Integration" and Section 4.4.2 discuss clinical implementation
Model performance	16	D;V	Report performance measures (with CIs) for the prediction model.	Section 3.7 and Table 1
Model-updating	17		If done, report the results from any model updating (i.e., model specification, model performance).	N/A – Development study
**Discussion**				
Limitations	18	D;V	Discuss any limitations of the study (such as nonrepresentative sample, few events per predictor, missing data).	Section 4.6 "Study Limitations and Methodological Considerations"
Interpretation	19a		For validation, discuss the results with reference to performance in the development data, and any other validation data.	N/A – Development Study
	19b	D;V	Give an overall interpretation of the results, considering objectives, limitations, and results from similar studies, and other relevant evidence.	Section 4 "Discussion"
Implications	20	V	Discuss the potential clinical use of the model and implications for future research.	Section 4.7 "Future Research Directions" and Section 4.4 "Clinical Implementation Considerations"
**Other information**				
Supplementary information	21	D	Provide information about the availability of supplementary resources, such as study protocol, Web calculator, and data sets.	"Data Availability Statement"
Funding	22	D	Give the source of funding and the role of the funders for the present study.	Funding

* Items relevant only to the development of a prediction model are denoted by D, items relating solely to a validation of a prediction model are denoted by V, and items relating to both are denoted D;V. We recommend using the TRIPOD Checklist in conjunction with the TRIPOD Explanation and Elaboration document.

Updated on January 22, 2026
