# Supplementary material for: Machine learning prediction of oxygen therapy in pediatric Mycoplasma pneumoniae pneumonia
Source: Front Digit Health. 2026 Feb 19;8:1755878. doi: 10.3389/fdgth.2026.1755878 (PMC12960568; doi:10.3389/fdgth.2026.1755878)
Supplement: Supplementary file 2 [file Table1.pdf]

Supplementary Tables

Table S1: Hyperparameteres that have been optimized for each algorithm

Model	Hyperparameters Tuned	Search Range	Rationale for Constraints
**Logistic Regression**	C, penalty	C: [0.1, 1.0, 10.0]; penalty: ['l2']	Moderate regularization range prevents both underfitting and overfitting
**Random Forest**	n_estimators, max_depth, min_samples_split	n_estimators: [100, 200, 300]; **max_depth: [5, 10, 15]**; min_samples_split: [2, 5, 10]	**Depth capped at 15** to prevent memorization with n=206
**XGBoost**	learning_rate, max_depth, n_estimators, subsample	learning_rate: [0.01, 0.05, 0.1]; **max_depth: [3, 4, 5]**; n_estimators: [100, 200, 300]; subsample: [0.7, 0.8, 0.9]	**Shallow trees (≤5)** appropriate for small datasets; conservative learning rates
**SVM**	C, kernel, gamma	C: [0.1, 1.0, 10.0]; kernel: ['rbf', 'linear']; gamma: ['scale', 'auto']	Moderate C range; 'scale' gamma adapts to feature variance
**KNN**	n_neighbors, weights	n_neighbors: [3, 5, 7, 9]; weights: ['uniform', 'distance']	k values appropriate for n=206 ($\sqrt{n} \approx 14$, used k≤9)
**MLP**	hidden_layer_sizes, learning_rate_init, alpha	hidden_layer_sizes: **(100,) only**; learning_rate_init: [0.001, 0.01]; alpha: [0.0001, 0.001]	**Single hidden layer enforced**; L2 regularization prevents overfitting
**LightGBM**	num_leaves, learning_rate, max_depth, feature_fraction	num_leaves: [15, 31, 50]; learning_rate: [0.01, 0.05, 0.1]; **max_depth: [3, 4, 5]**; feature_fraction: [0.6, 0.8, 1.0]	**Shallow trees and limited leaves** for small sample size

**Naive Bayes**	var_smoothing	var_smoothing: [1e-12, 1e-9, 1e-6]	Minimal tuning; inherently simple model
**TabTransformer**	n_steps, gamma, n_d, n_a	n_steps: [3, 5, 7]; gamma: [1.0, 1.5, 2.0]; n_d/n_a: [16, 32, 64]	**Reduced architecture** (3-7 layers vs. typical 10+) for tabular data with small n
